# Venereal Memoranda

**Published:** 1886-02

**Authors:** 


**Venereal Memoranda.** A Manual for the Student and Practitioner. By P. A. MORROW, Clinical Professor of Venereal Diseases in the University, City of New York, etc., etc. New York: Wm. Wood & Co. 1885.

We have had occasion to commend Wood's Pocket Manuals, and these two are peculiarly good. Both of them contain a vast amount of information on their respective subjects, "boiled down," so to speak, but the essential facts and principles are presented in a way convenient for the student, and even for the practitioner.

